# Impact evaluation of an interdisciplinary approach to patients with chronic non-cancer pain in Chilean primary care

**DOI:** 10.1186/s12913-025-12560-9

**Published:** 2025-03-24

**Authors:** Paula Zamorano, Teresita Varela, Isidora Salvatierra, Alvaro Tellez, Manuel Espinoza, Gustavo Torres, Victoria Rodríguez, María José Figueroa, Alejandro Rodríguez, Denisse Figueroa, Leonardo Silva, Sheila Salazar, Víctor Lucero, Francisco Suarez

**Affiliations:** 1 Innovación ANCORA UC, Facultad de Medicina, Pontificia Universidad Católica de Chile, Santiago, Chile; 2 Escuela de Ciencias de la Salud, Pontificia Universidad Católica de Chile, Santiago, Chile; 3 Escuela de Salud Pública, Pontificia Universidad Católica de Chile, Santiago, Chile; 4 Departamento de Medicina Familiar, Pontificia Universidad Católica de Chile, Santiago, Chile; 5 Unidad de Dolor, Pontificia Universidad Católica de Chile, Santiago, Chile; 6 Unidad de Dolor, Hospital de La Florida, Santiago, Chile; 7 Centro de Salud Familiar Maffioletti, Corporación Municipal de La Florida, Santiago, Chile; 8 Centro de Salud Familiar La Florida, Corporación Municipal de La Florida, Santiago, Chile; 9 Unidad de Análisis y Gestion de Información en Salud, Servicio de Salud Metropolitano Sur Oriente, Santiago, Chile; 10 Present address: Santiago, Chile

**Keywords:** Non-cancer chronic pain, Multimorbidity, Primary care, Patient-centered care

## Abstract

**Introduction:**

Chronic non-cancer pain affects one-third of the global population. In Chile, its prevalence is estimated at 34%, surpassing the prevalence of diabetes mellitus and hypertension. Its high costs reveal that clinical treatment causes the greatest economic impact, followed by days of work absenteeism.

**Objective:**

This study aims to evaluate the impact on resource consumption, quality of life, and pain perception in patients with CNCP, an interdisciplinary approach implemented in Chilean primary care public health.

**Methods:**

A concurrent cohort study was conducted with patients aged 25 to 64 with chronic non-cancer musculoskeletal pain. The population studied was 698 patients receiving primary health services in centers with similar size and territorial proximity. The clinical intervention introduced patient-centered care, psychotherapy and physiotherapy from the perspective of the neuroscience of pain. The impact analysis was conducted using negative binomial regression models, generalized linear models, and ordered logistic regressions.

**Results:**

Results show that the patients who were intervened increased the number of physician consultations at primary care (IRR: 1.56; 95% CI 1.30–1.87) and increased medication consumption (coef 2.38; 95% CI 2.10–2.67) compared to control patients. Intervened patients improved their quality of life (COEF 0.14; 95% CI 0.09–0.19), and pain perception was statistically significant. Despite the health system’s structural, cultural, and organizational barriers, the intervention was implemented and consolidated in daily operation, providing learnings for a further scale-up.

**Conclusion:**

The study demonstrates that an interdisciplinary approach to chronic non-cancer pain management in Chilean primary care improves quality of life and pain perception while increasing healthcare resource use. Despite system barriers, the intervention was successfully implemented and sustained within patient-centered care. These findings highlight the need for resource reallocation to ensure long-term sustainability and scalability through the public health system.

**Supplementary Information:**

The online version contains supplementary material available at 10.1186/s12913-025-12560-9.

## Introduction

Chronic non-cancer pain (CNCP) is a public health challenge, affecting approximately one-third of the global population [1, 2]. Pain is multifactorial in its origin and has physical, psychological, and social repercussions for the individual and the healthcare system [3] which makes it challenging to manage effectively [4, 5]. Most healthcare systems rely heavily on pharmacological treatments, despite growing concerns over opioid dependency and limited long-term effectiveness [6]. On the other hand, access to non-drug interventions, such as physiotherapy and psychological support, is often restricted due to financial and structural barriers [7]. These challenges result in increased healthcare costs, reduced quality of life for patients, and economic burdens caused by productivity losses and work absenteeism [8].

To address these issues, countries have implemented interdisciplinary approaches that integrate medical, psychological, and rehabilitation interventions [9, 10]. In Europe, the Joint Action CHRODIS-PLUS initiative has developed an Integrated Multimorbidity Care Model to improve coordination and continuity of care for patients with multimorbidity [11]. Similarly, pilot programs targeting frail older adults have shown that individualized care plans, case management, and patient education can lead to improved pain management outcomes [12]. In the United States, interdisciplinary pain clinics have adopted interdisciplinary treatment strategies, combining pharmacological, physical, and cognitive-behavioral therapies to enhance patient outcomes [13, 14]. While these models have demonstrated effectiveness, their implementation and scalability depend on healthcare system resources, policy support, and provider training.

Despite evidence supporting interdisciplinary approaches [10] significant gaps persist between research findings and policy implementation [15, 16]. Guidelines advocate for multimodal pain management; however, health systems often struggle to operationalize these recommendations due to funding constraints, workforce shortages, and rigid care structures [17]. In Chile, the CNCP approach is primarily delivered through primary healthcare (PHC), which offers medical and medication and community rehabilitation. A recent study revealed a prevalence of CNCP of 34%, whereas 20% suffer from musculoskeletal pain [18]. The economic burden represents an annual national cost of USD 1.3872 billion for musculoskeletal CNCP, equivalent to 0.417% of the Gross Domestic Product. The Chilean healthcare system’s current response to these individuals’ needs entails a high cost from frequent health services utilization [8]. Other studies on costs, productivity losses, and public policy proposals have also demonstrated the magnitude of the problem [8, 16], raising awareness to mobilize the ministry on the issue.

The [8, 16, 18–29] study aims to evaluate the impact of an interdisciplinary approach—incorporating patient-centered care, physiotherapy, and psychotherapy—on resource consumption, quality of life, and pain perception in CNCP patients of Chile’s public primary healthcare public system. By assessing feasibility and effectiveness, the study seeks to contribute to the ongoing discussion [10] on scalable and sustainable CNCP management strategies.

## Methodology

A cohort pilot study was conducted to compare the intervention group with the non-intervention group from two intervention centers and two control centers. The non-intervention group received the usual standard care based on medical consultations and medication treatment in PHC. Centers were selected based on territorial proximity and the health center’s size. The follow-up period was from July 2022 to July 2024, during which anonymized data was collected from the health service’s Analysis and Information Management Unit (UNAGIS) for impact evaluation.

### Population

The target population was selected according to age and diagnostic inclusion criteria as follows: adults (≥ 25 years and < 65 years) with musculoskeletal CNCP (lumbar pain, fibromyalgia, coxarthrosis, Knee arthrosis, shoulder pain, and/or rheumatoid arthritis). Patients with the following diagnoses were excluded: palliative care, active cancer, stage 5 chronic kidney disease, severe physical dependence, and alcohol and drug addiction, as the health system already offers a service portfolio in PHC. Healthcare professionals carried out the selection process during clinical check-ups. 75% of people with CNCP attended by healthcare professionals were eligible for the intervention. A total of 400 individuals received the intervention, which was compared with 298 control individuals. A matching process based on age and sex was conducted for the impact analysis. The patient dropout rate during the study was 16%.

### Clinical intervention

**Fig. 1:**
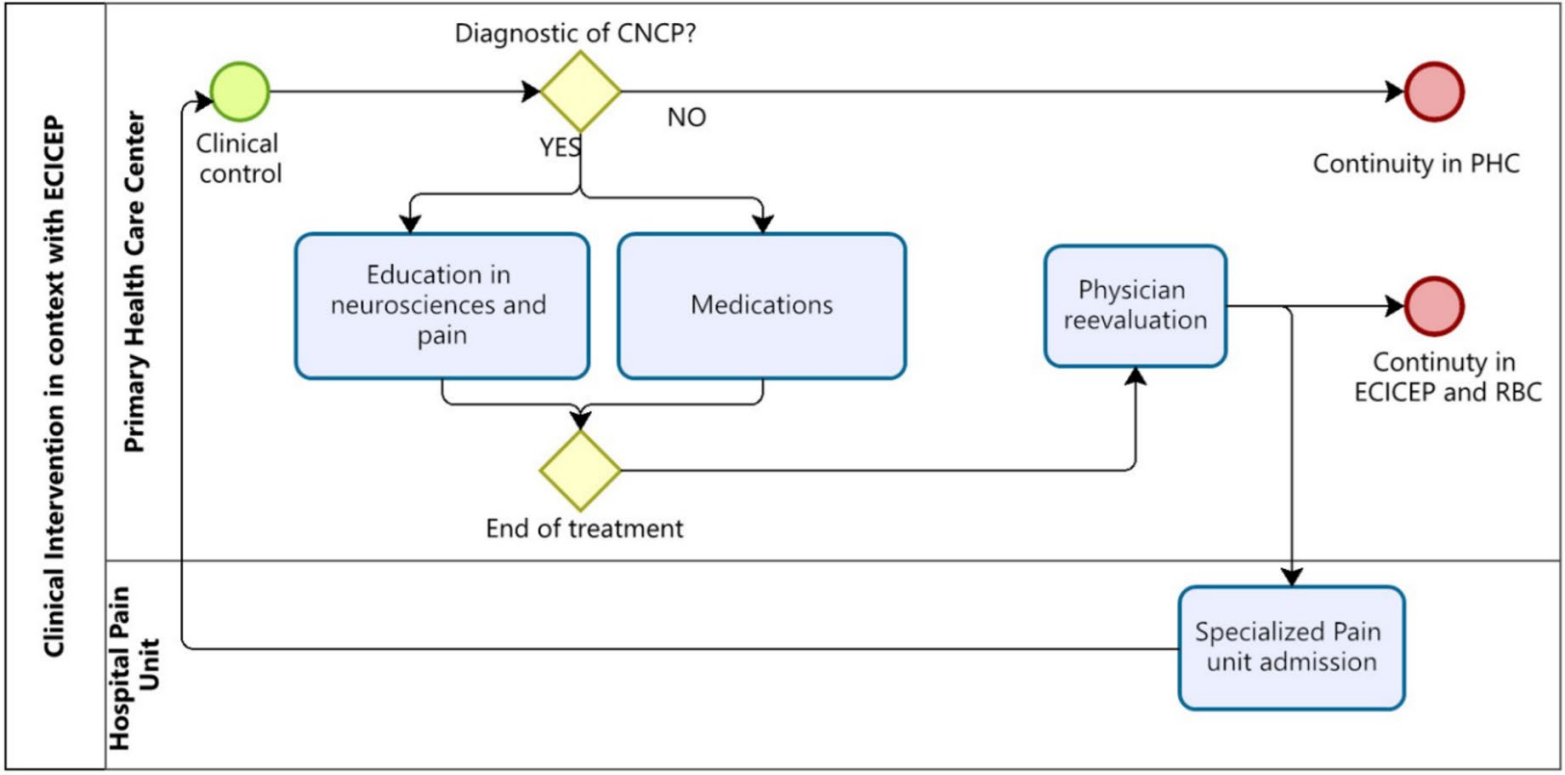
Clinical intervention for non-cancer chronic pain. Source: Own elaboration. ECICEP: Comprehensive Care Strategy for people with multimorbidity [20]. RBC: Community based rehabilitation. Source: Own elaboration. ECICEP: Comprehensive Care Strategy for people with multimorbidity [20]. RBC: Community based rehabilitation

The clinical intervention is based on diversifying and expanding existing services in PHC through interdisciplinary care and access to an expanded pharmaceutical basket, as described in the technical guidelines of the Chilean Ministry of Health [19]) (Fig. 1).

Enrollment in the pilot program begins with a primary health care (PHC) clinical check-up with a physician or other healthcare professionals (for individuals already diagnosed with non-oncological chronic pain). After this evaluation, the patient accesses services based on what is agreed upon in their comprehensive care plan. Each patient receiving the intervention was assessed using the EuroQol-5D (EQ-5D) and the Pain Analog Rating Scale (VAS) [21] at enrollment and again three months after the intervention began to detect changes in pain assessment and health-related quality of life.

### Clinical follow-up - neuroscience education and psychoeducation for the treatment of non-oncological chronic pain based on neuroscience and psychoeducation of pain

**Fig. 2:**
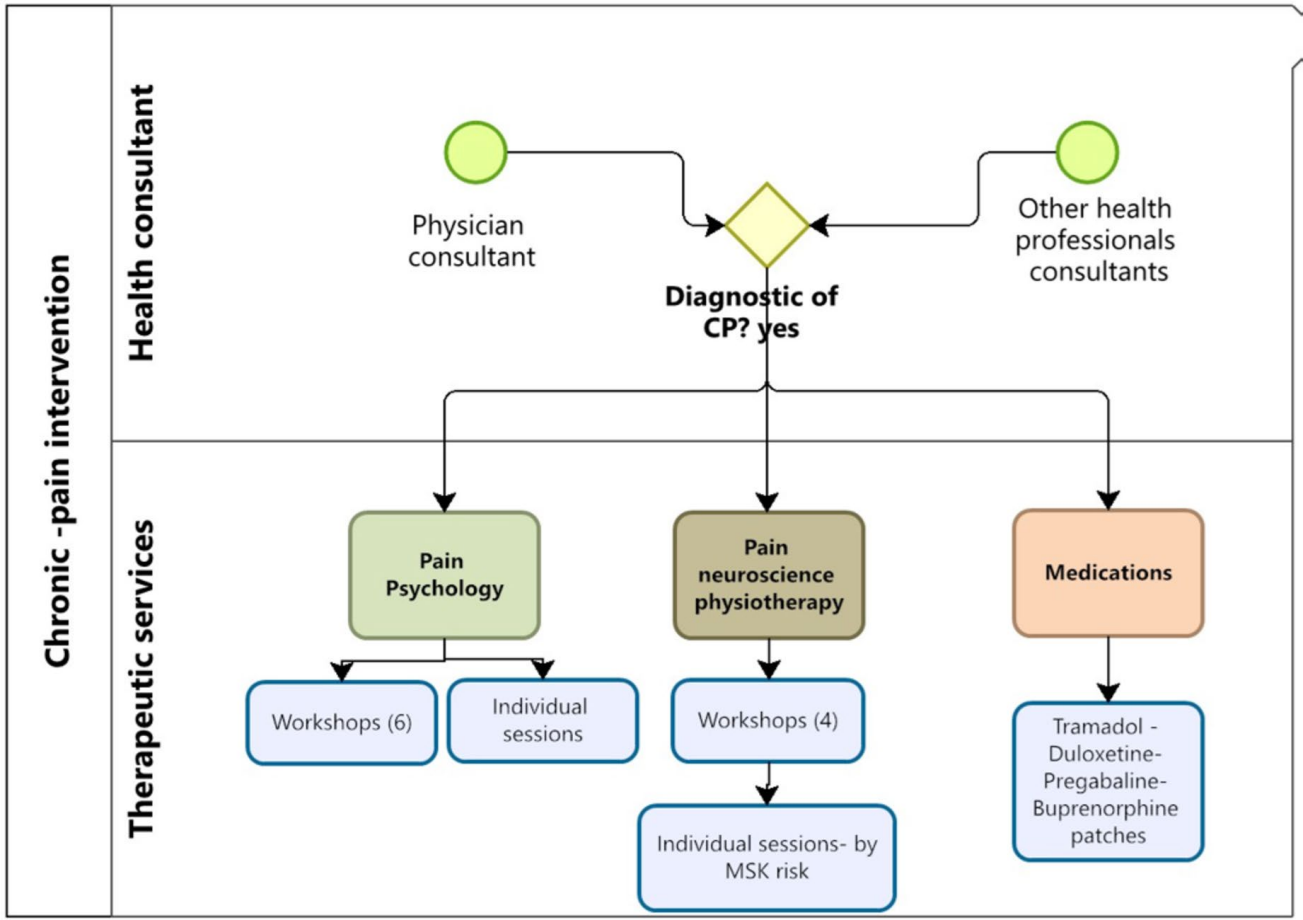
Organization of workshops based on education in neurosciences and pain psychoeducation and individual sessions for patients with chronic non-cancer pain. Source: Own elaboration

The new physical therapy and psychology services are described, which enabled the diversification of the current portfolio available for people with Chronic non-cancer pain in PHC (Fig. 2).

#### Pain psychology

A psychologist led psychoeducational group sessions focused on educating participants about relaxation techniques, identifying thoughts and emotions related to pain, and understanding how these factors affect various areas of a person’s life, their context, pain triggers, characteristics, and causes, among other activities. The sessions lasted 60 min each; they included an introduction to cognitive-behavioral therapy, breathing and relaxation training, attention management, cognitive restructuring, emotion management, assertiveness, problem-solving, time management, and reinforcement activities (Table 1).

#### Physiotherapy based on pain neuroscience education

It begins with applying the Start MSK Screening tool [22] and the Pain Analog Rating Scale (VAS). The treatment emphasizes a comprehensive understanding of pain as a complex physical, psychological, and social condition. It is based on four pillars: pain education, therapeutic exercise, sleep management, and stress management. The treatment plan is divided between individual (30 min each) and group interventions (60 min each) (Table 1) depending on the Start MSK Screening tool risk level, with higher-risk patients receiving more individual sessions.

**Table 1:** List of group psychoeducational and physiotherapy workshops for people with chronic non-cancer pain

Educational Area	Workshop	Objectives
Pain Psychotherapy	Introduction to Basic Pain Concepts	Provide basic knowledge about chronic pain.
	Emotions Associated with the Pain Experience	Facilitate the identification of emotions and their connection to somatic and pain experiences. Train in relaxation techniques.
	The Process of Active Acceptance	Facilitate the identification of the role of anxiety in the fight/flight response to discomfort and its effects. Introduce active acceptance as an alternative to control.
	Attention Focus	Link pain perception to hyper-alertness and vigilance over bodily sensations. Emphasize the importance of maintaining flexible attention. Train in meditation and body visualization.
	Communication	Raise awareness of how pain issues affect communication patterns and may lead to isolation. Connect with personal needs. Propose assertive communication alternatives.
	Recap and Relapse Prevention	Encourage an active role in maintaining learned skills. Identify potential barriers and obstacles to maintaining progress. Prevent relapses.
Physiotherapy workshops with a focus on pain neuroscience education	Pillars of Physiotherapy Treatment for Persistent Pain	Understand why pain education has positive effects. Identify personal beliefs and experiences influencing pain perception. Understand therapeutic exercises tailored to reduce pain and improve quality of life.
	Nervous System as an “Alarm System”	Through metaphors, understand the complexity of the pain perception system and how its operation influences painful experiences. Understand the phenomenon from a multifactorial perspective. Learn how different factors can influence pain perception and highlight some tools to manage these factors.
	Sleep Hygiene and its Relationship to Pain	Understand the importance of sleep in managing persistent pain and its bidirectional relationship with sleep deprivation and increased nervous system sensitivity. Provide sleep hygiene counselling and offer strategies to improve sleep quality.
	Therapeutic Exercise and Self-Efficacy	Understand the effectiveness of therapeutic exercise in reducing pain and improving functionality. Propose individualization and gradualness in the exercise prescription. Promote self-efficacy and confidence in performing the exercise. Provide specific therapeutic exercises that may be helpful in the initial phases of treatment.

Source: Own elaboration

#### Individual physiotherapy sessions

Therapeutic exercise, individualized and gradual, is presented as key to reducing pain and improving quality of life. The relationship between sleep and pain is addressed, highlighting the importance of sleep hygiene. The sessions seek to empower patients, making them active agents in their rehabilitation. The treatment plan consists of individual and group interventions depending on the risk:

- Low risk: 4 workshops sessions only as described in Table 1.
- Medium risk: 4 group work sessions as described in Table 1 plus 6 individual sessions (education in pain neuroscience, graded activity/exercise and promotion of healthy lifestyle habits.
- High risk: 4 group work sessions as described in Table 1 plus 10 individual sessions (education in pain neuroscience, symptom management exercises, graded exposure and promotion of healthy lifestyle habits).

Access to Medications. Restrictions on the quantity and limitations related to GES health conditions for prescribing tramadol and paracetamol were removed. Pregabalin, duloxetine, and buprenorphine patches were added to the PHC medication arsenal.

#### Reevaluation

Once the proposed intervention cycle is completed, the patient can continue treatment in primary care, undergo community rehabilitation, or be referred to a specialized level, depending on the case.

#### Referral to pain units in specialized centers

The study established the following referral criteria from PHC to specialized secondary pain units. After having accesssed the services mentioned above, if the patient remains with a VAS greater than 6, if there is still diagnostic doubt about the type of pain, or if there has not been a 2-point improvement in VAS, the patient meets the criteria to be referred to the secondary level.

### Implementation process

The implementation process was carried out in three stages. First, the clinical teams received induction on the intervention, its objectives, requirements, co-creation aspects, and expected outcomes. Healthcare professionals were assigned for the intervention and completed courses on interdisciplinary chronic pain management along with internships to apply the new knowledge and tools daily. The second stage involved clinical activities, biweekly clinical consultations, and expert case reviews organized and supervised by the ANCORA UC Innovation teams. The final stage focused on data collection, concluding the pilot, distributing workshop materials, and consolidating the intervention for future national replication.

### Data analysis

The impact of the intervention was evaluated in terms of (1) the number of visits to primary emergency services, (2) the number of visits to hospital emergency services, (3) the number of medical consultations in PHC, (4) the cost of medication consumption; (5) days of medical leave; and health-related quality of life (HRQoL) measured by EuroQol-5D (EQ5D) [23] and a specific pain question from the EQ5D questionnaire. The following adjustment variables were studied to estimate the effect: sex, age, number of comorbidities, diagnosis, insurance, and resource use according to ACG [24]. A univariate analysis was performed, with chi-square tests for discrete variables and t-tests for continuous variables. The effect analysis used negative binomial regression models, generalized linear models, and ordered logistic regressions. The statistical analysis was carried out in Stata 14.

## Results

The baseline characteristics of the target population are presented in Table 2. The only variable that showed statistically significant differences was age.

**Table 2:** Population’s baseline characteristics

	Intervention (*n* = 400)		Control (*n* = 298)		*p*-value
Sex (Feminine) n %	342 (86%)		261 (87.6%)		< 0.704
Age mean (± SD)	50.3 (8.2)		55.1 (8.5)		< 0.001
Number of comorbidities (P25-P75)	1–5		1–6		< 0.023
Insurance category					< 0.177
FONASA A	87	21.8%	61	20.5%	
FONASA B	109	27.3%	86	29%	
FONASA C	82	20.5%	51	17.2%	
FONASA D	91	22.8%	80	27%	
Not informed	30	7.5	15	5.05%	
Private	1	0.25%	4	1.4%	
Resource utilization band (RUB-ACG)					< 0.210
0	29	9.18%	25	10.29%	
1	23	7.28%	11	4.53%	
2	26	8.23%	24	9.88%	
3	173	54.75%	128	52.67%	
4	42	13.29%	45	18.52%	
5	23	7.28%	10	4.12%	

*FONASA* Fondo nacional de salud, public insurance, *ACG* Adjusted clinical groups. Source: own elaboration

### Health services utilization results

The study shows that intervened patients have a 1.5 times higher risk of visiting primary care emergency services than control patients, which is statistically significant (Table 3). However, they have a lower risk of visiting hospital emergency services. Regarding medical consultations at PHC, intervened patients have a 3.5 times higher risk of consulting than control patients, which is also statistically significant.

It is important to note that, when analyzing medical consultation data from the year before the intervention, those who already had more frequent consultations continued to have more visits than those who previously consulted less. Medication consumption among intervened patients is 2.38 times higher (refer to supplementary materials for details on consumption), with a 24% increase in days of medical leave. However, this increase is not statistically significant.

Regarding quality-of-life and pain perception results, health status was analyzed using the Chilean EQ5D [21] validation. The intervention is significantly associated with a 14% improvement in quality of life (Coef: 0.14; 95% CI 0.09–0.19). Regarding question four of the EQ5D questionnaire about pain/discomfort, the intervened patients initially experienced more pain than the controls. The intervention is significantly associated with a greater reduction in pain and a lower likelihood of experiencing severe pain (patients with mild pain are less likely to progress to moderate pain, and those with moderate pain are less likely to progress to severe pain). The results of patient pain reported through the VAS demonstrate that intervened patients have statistically significantly decreased their rest pain by 11% compared to non-intervened patients. However, no significance was found in the decrease in the pain perceived during exercise.

**Table 3:** Results of the impact analysis

Variable	Estimator	(95% CI)	*p*-value
Number of emergency visits to PHC (SAPU-SAR)	1.57 (IRR)	(1.30–1.87)	< 0.001
Number of hospital emergency visits	0.87 (IRR)	(0.71–1.96)	< 0.191
Number of medical consultations in PHC	3.55 (IRR)	(3.10–4.07)	< 0.001
Total medication cost	2.38 (coef)	(2.10–2.67)	< 0.001
Health-related quality of life	0.14 (coef)	(0.09–0.19)	< 0.001
Health-related quality of life– question 4 Pain/Discomfort	0.28 (coef)	(0.18–0.43)	< 0.001
Analog numerical pain (ANP) scale at rest (VAS)	0,11 (coef)	(0,02–0,20)	< 0.021
Analog numerical pain scale (ANP) during activity (VAS)	0,01 (coef)	(0,10–0,70)	< 0.748

SAPU: primary care emergency service; SAR: Resolution Urgent Primary Care Services. VAS: Analog numerical pain scale. Baseline value for Health quality of life is 0.43

### Implementation results

During the pilot phase, adjustments were made to the schedules, new activities were introduced, and clinical records protocols were defined. The priority was placed on delivering comprehensive care to those individuals identified as being at the highest risk according to the ECICEP framework. Regarding medication management, processes for the procurement and dispensation of additional medications, particularly opioids, were established. In addition, physical spaces were reserved for conducting the workshops. The total cost of human resources assigned to the intervention was estimated at USD 2,000 per month, covering 22 weekly hours of physiotherapy, 11 weekly hours of psychological support, and 11 weekly hours of pilot coordination.

Regarding change management, the ANCORA UC Innovation team provided weekly support to the healthcare teams at each CESFAM. This support focused on operational aspects, addressing gaps in human capital, enhancing communication strategies, and fostering an interdisciplinary, patient-centered approach aligned with the ECICEP model. A key element in biweekly clinical consultations was the agreement on a personalized care plan, which enabled patients to better understand the rationale behind their referrals to specific services. To support the training of health teams, technical conferences and UC courses were organized to enhance the skills of primary care professionals. Clinical modeling and medication prescription training were also provided by the physiatry team at Hospital de La Florida.

## Discussion

The study evaluated an interdisciplinary approach integrating physiotherapy, psychotherapy, and patient-centered care to manage chronic non-cancer pain in Chile’s public primary healthcare network. The intervention aimed to improve resource efficiency, reduce pain, and enhance patients’ quality of life. Results showed a significant reduction in pain perception and a 14% improvement in health-related quality of life, though healthcare utilization increased, particularly in primary care consultations and medication prescription. The study highlights the feasibility of a multidisciplinary CNCP management model, emphasizing the need for policy support and continuously work on operational, structural, and cultural conditions, as demonstrated in previous pilots on multimorbidity [26].

The results demonstrate interdisciplinary approach to chronic non-cancer pain (CNCP) in primary healthcare settings significantly enhances patients’ quality of life. The integration of physiotherapy and psychological interventions, alongside pharmacological management, contributed to an observed improvement in health-related quality of life. By addressing both the physical and psychological aspects of pain, this model facilitated better coping mechanisms and functional recovery. Moreover, the reduction in severe pain experiences, as indicated by the EQ-5D pain/discomfort dimension, and pain perception with VAS demonstrate the benefit from comprehensive management as already shown in multimorbidity setting in Chile and other countries [27, 28]. The intervention successfully mitigated pain intensity through patient-centered strategies such as pain neuroscience education, cognitive-behavioral therapy, and graded exercise programs. Notably, while rest pain showed improvement, there was no significant reduction in activity-related pain, indicating potential challenges in addressing movement-related discomfort. This highlights the need for further refinement in the intervention to optimize therapeutic exercise and functional rehabilitation to target pain experienced during activity more effectively. Despite this limitation, these findings underscore the importance of multimodal care approaches in improving the daily lives of individuals with chronic pain.

Health services utilization results reveals that implementing national and international evidence involves higher clinical consultants and drugs consumption. The findings of this study align with prior research, such as Espinoza et al. [8] in Chile, which identified therapeutic approaches as the primary cost driver in chronic pain management. Patients in the intervention group demonstrated increased engagement with primary healthcare services, reflecting the broader trend that improved health services variety and patient awareness can lead to higher healthcare utilization and medical leave notifications. However, this increase raises questions about healthcare system capacity and whether such models can be sustainably integrated into routine practice.

While the study did not explicitly quantify changes in medication use, it was observed an increase in prescription of analgesics, including opioids and nonsteroidal anti-inflammatory drugs (NSAIDs). This finding is likely due to the fact that, before the implementation of the intervention, patients incurred out-of-pocket expenses to acquire them outside the public health system [29]. Currently, these medications are provided directly in their health centers, which could explain the increase observed in our study. For example, patients in the control group do not report consuming duloxetine, pregabalin, or buprenorphine patches, which highlights gaps in access to these treatments. On one side it is positive that the intervention avoids out-of-pocket expenditure, but on the other side, the study expected an inferior increase in mediations costs. Future research should examine how interdisciplinary care impacts long-term medication adherence and whether it leads to a reduction in unnecessary pharmacological treatments minimizing the risks associated with prolonged drug use and overburdened healthcare services.

The main strengths of the study are the completeness of the data analysed can be mentioned since the resource consumption of each individual in the primary, secondary and tertiary network was incorporated. Also, the study was conducted in a real context, which are fundamental for implementing public policies. Regarding limitations, the convenient selection of the intervened centers is a limitation, which was addressed by choosing control centers of similar size and territorial proximity. The intervention group had a higher baseline severity of pain compared to controls, which may have influenced the observed effect sizes. Additionally, the study relied on self-reported measures, which are inherently subject to bias and variability. Another limitation is the relatively short follow-up period of three months post-intervention, which may not capture long-term outcomes or potential relapse in pain perception and quality of life.

In conclusion, this study highlights the benefits of an interdisciplinary approach to managing chronic non-cancer pain (CNCP) in primary healthcare, showing significant improvements in patients’ quality of life and pain perception. The integration of physiotherapy, psychology, and pharmacological management improved quality of life and reduced pain perception. The successful implementation and potential for scaling this model within the ECICEP framework depend on the proper training of healthcare teams, effective change management, and the reallocation of resources to ensure the efficient functioning of the health system. While the intervention shows promising potential in improving patient outcomes, further research is necessary to evaluate its long-term impact and sustainability.

## Supplementary Information

Supplementary Material 1

## Data Availability

Data is provided within the manuscript or supplementary information files.

## References

[1] Croft M, Mayhew R. Prevalence of chronic non-cancer pain in a UK prison environment. Br J Pain. 2015;9(2):96–108 [cited 2025 Jan 30]. Available from: https://pubmed.ncbi.nlm.nih.gov/26516564/. 26516564 10.1177/2049463714540895 PMC4616963

[2] Cáceres-Matos R, Gil-García E, Barrientos-Trigo S, María A. Consecuencias del Dolor Crónico no Oncológico en la edad adulta. Scoping review. Rev Saude Publica. 2020;54(39):1–13 Available from: http://www.rsp.fsp.usp.br/. 10.11606/s1518-8787.2020054001675 PMC7135143 32321056

[3] Cohen SP, Vase L, Hooten WM. Chronic pain: an update on burden, best practices, and new advances. Lancet. 2021;397(10289):2082–97 [cited 2024 Sep 29]. Available from: https://pubmed.ncbi.nlm.nih.gov/34062143/. 34062143 10.1016/S0140-6736(21)00393-7

[4] Tauben DSRB. Pharmacologic management of chronic non-cancer pain in adults. UpToDate. 2021; Available from: https://bit.ly/3qX9Irb.

[5] Workman EA, Hubbard JR, Felker BL. Comorbid psychiatric disorders and predictors of pain management program success in patients with chronic pain. Prim Care Companion J Clin Psychiatry. 2022;4(4):137 [cited 2021 Oct 7]. Available from: https://pubmed.ncbi.nlm.nih.gov/15014721/, 10.4088/pcc.v04n0404. 10.4088/pcc.v04n0404 PMC315482 15014721

[6] Genova A, Dix O, Thakur M, Sangha PS. Chronic non-cancer pain management and addiction: a review. Cureus. 2020;12(2). Available: https://pubmed.ncbi.nlm.nih.gov/32076590/, 10.7759/cureus.6963 10.7759/cureus.6963 PMC7015117 32076590

[7] Dickson C, de Zoete RMJ, Berryman C, Weinstein P, Chen KK, Rothmore P. Patient-related barriers and enablers to the implementation of high-value physiotherapy for chronic pain: a systematic review. Pain Med. 2024;25(2):104–15. 10.1093/pm/pnad134. [cited 2025 Jan 30] 37769242 10.1093/pm/pnad134 PMC10833081

[8] Espinoza MA, Bilbeny N, Abbott T, Carcamo C, Zitko P, Zamorano P et al. Cost analysis of chronic pain due to musculoskeletal disorders in Chile. PLoS One. 2022;17(10). [cited 2022 Dec 13]. Available from: https://pubmed.ncbi.nlm.nih.gov/36301984/. 10.1371/journal.pone.0273667 PMC9612497 36301984

[9] Danilov A, Danilov A, Barulin A, Kurushina O, Latysheva N. Interdisciplinary approach to chronic pain management. Postgrad Med. 2020;132(sup3):5–9. Available: https://pubmed.ncbi.nlm.nih.gov/32298161/. 32298161 10.1080/00325481.2020.1757305

[10] Montero-Cuadrado F, Barrero-Santiago L, Santos-Bermejo M. Pain revolution in the public health system: Active coping strategies for chronic pain unit. Braz J Phys Ther. 2025;29(2):101176. Available from: https://linkinghub.elsevier.com/retrieve/pii/S1413355525000061. 10.1016/j.bjpt.2025.101176 PMC11833345 39892286

[11] Palmer K, Marengoni A, Jureviviene E, Laatikainen T, Mammarella F, Muth C et al. Multimorbidity care model: recommendations from the consensus meeting of the joint action on Chronic Diseases (CHRODIS). 2016;(February):1–21. 10.1016/j.healthpol.2017.09.006 10.1016/j.healthpol.2017.09.006 28967492

[12] Swedish Agency for Health Technology Assessment and Assessment of Social Services. Multimodal and interdisciplinary interventions for long term pain. 2021. [cited 2025 Jan 30]. Available from: https://www.ncbi.nlm.nih.gov/books/NBK583205/. 35998243

[13] Schwan J, Sclafani J, Tawfik VL. Chronic pain management in the elderly. Anesthesiol Clin. 2019;37(3):547–60. [cited 2025 Jan 30]. Available from: http://www.anesthesiology.theclinics.com/article/S1932227519300424/fulltext. 10.1016/j.anclin.2019.04.012 PMC6658091 31337484

[14] Nahin RL. Use of Multimodal Multidisciplinary Pain Management in the US. JAMA Netw Open. 2022;5(11):e2240620–e2240620. [cited 2025 Jan 30]. Available from: https://jamanetwork.com/journals/jamanetworkopen/fullarticle/2798137. 10.1001/jamanetworkopen.2022.40620 36342720

[15] Espinoza M, Repetto P, Cabieses B, Varagas C, Zitko P. Capítulo I - Propuesta de política pública para el manejo del dolor crónico musculoesquelético en Chile. Propuestas para Chile - Concurso Políticas públicas. 2017;2017:19–42. Available: https://politicaspublicas.uc.cl/publicacion/capitulo-i-propuesta-de-politica-publica-para-el-manejo-del-dolor-cronicomusculoesqueletico-en-chile/.

[16] Varela T, Zamorano P, Mha PT, Rodriguez MV, Espinoza M. Integrating noncancer chronic pain to Multimorbidity: A real practice challenge in Chile. Value Health Reg Issues. 2023;38:45–6. 10.1016/j.vhri.2023.05.008. 37467539 10.1016/j.vhri.2023.05.008

[17] Leonard C, Ayele R, Ladebue A, McCreight M, Nolan C, Sandbrink F, et al. Barriers to and facilitators of multimodal chronic pain care for veterans: A National qualitative study. Pain Med. 2021;22(5):1167–73. 10.1093/pm/pnaa312. [cited 2025 Jan 30] 32974662 10.1093/pm/pnaa312

[18] Miranda JP, Quezada P, Caballero P, Jiménez L, Morales A. Bilbeny, Norberto; Vega JC. Dolor Crónico En Chile. Revista Médica Clínica Las Condes. 2019;30(6):397–406.10.1016/j.rmclc.2019.08.002

[19] Ministerio de Salud; Gobierno de Chile. ORIENTACION TECNICA. Manejo del dolor cronico no oncologico en personas de 15 años y mas, en Atención Primaria. 2021. Available: https://www.ached.cl/storage/userfiles/files/Orientacion-Tecnica.pdf.

[20] Ministerio De Salud (MINSAL). Estrategia de Cuidado integral Centrado En Las personas Para La Promoción, prevención y Manejo de La Cronicidad En contexto de multimorbilidad. Ministerio De Salud. 2021;80. https://www.minsal.cl/wp-content/uploads/2021/06/Marco-operativo_-Estrategia-de-cuidado-integral-centrado-en-las-personas.pdf?utm_source=chatgpt.com.

[21] Olivares-Tirado P. Calidad de vida relacionada a la salud en población general 2005. 2006. [cited 2024 Sep 29]. Available from: https://www.supersalud.gob.cl/documentacion/666/articles-3818_recurso_1.pdf.

[22] Dunn KM, Campbell P, Lewis M, Hill JC, van der Windt DA, Afolabi E, et al. Refinement and validation of a tool for stratifying patients with musculoskeletal pain. Eur J Pain. 2021;25(10):2081–93. 10.1002/ejp.1821 34101299 10.1002/ejp.1821

[23] EQ-5D-5L| EuroQol. [cited 2024 Dec 4]. Available from: https://euroqol.org/information-and-support/euroqol-instruments/eq-5d-5l/.

[24] ACG System Excerpt from Technical Reference Guide. 2009;(December):0–41. https://www2.gov.bc.ca/assets/gov/health/conducting-health-research/data-access/johns-hopkins-acgsystem-technical-reference-guide.pdf.

[25] Ministerio de Salud. Subsecretaria de Redes Asistenciales. Estrategia de cuidado integral centrado en las personas para la promoción, prevención y manejo de la cronicidad en contexto de multimorbilidad. 2020.

[26] Zamorano P, Varela T, Tellez A, Espinoza M, Munoz P, Suarez F. Impact of a patient-centered care model implemented in public health facilities in Chile: A real world evidence evaluation. J Public Health Epidemiol. 2022;14(1):1–9. 10.5897/JPHE2021.1371

[27] Mery Bolívar Vargas P, Zamorano JB. Colombia - Proposal for a Comprehensive Healthcare Management Model for People with Multimorbidity and their Caregivers. 2024. [cited 2024 May 9]. Available from: http://documents.worldbank.org/curated/en/099040824130512058/P1706381eb101d06d182571490e9db0e81d.

[28] Zamorano P, Muñoz P, Espinoza M, Tellez A, Varela T, Suarez F et al (2022) Impact of a high-risk Multimorbidity integrated care implemented at the public health system in Chile. PLoS ONE 17(1):e0261953. 10.1371/journal.pone.0261953 35030178 10.1371/journal.pone.0261953 PMC8759679

[29] Castillo-Laborde C, Dintrans PV. Caracterización del gasto de bolsillo en salud en Chile: una mirada a dos sistemas de protección. Rev Med Chil. 2013;141(11):1456–63. [cited 2024 Sep 29]. Available from: http://www.scielo.cl/scielo.php?script=sci_arttext&pid=S0034-98872013001100013&lng=es&nrm=iso&tlng=es. 10.4067/S0034-98872013001100013 24718473

