# Supplementary material for: Impact evaluation of an interdisciplinary approach to patients with chronic non-cancer pain in Chilean primary care
Source: BMC Health Serv Res. 2025 Mar 24;25:423. doi: 10.1186/s12913-025-12560-9 (PMC11931808; doi:10.1186/s12913-025-12560-9)

**Supplementary material**

**Description of medication consumption**

Table 1. Average cost by patient medication consumption by age group during the intervention period (in USD) (1 USD = 952.38 CLP)

| **Population** | **Age 25 to 45** | **Age 46 to 65** |
| --- | --- | --- |
| Exposed | 113,854.9 | 94,995.6 |
| Un-exposed | 1,048.5 | 4,695.3 |
| **Total medication consumption** | **75,951.9** | **55,937.8** |

Table 2. Average cost of patient medication consumption by sex during the intervention period (in USD) (1 USD = 952.38 CLP).

| **Population** | **Male** | **Female** | **Other** |
| --- | --- | --- | --- |
| Exposed | 100,520.8 | 99,481.5 | 35,922.2 |
| Un-exposed | 2,374.4 | 5,015.6 | 132.0 |
| **Total avergage medication consumption** | **61,746.9** | **58,593.3** | **23,140.0** |

Table 3. Increase in total medication consumption by diagnostic. The comparator is Arthritis rheumatoid.

| **Diagnostic** | **Coef.** | **Std. Err.** | **t** | **P>|t|** | **[95% Conf.** | **Interval]** |
| --- | --- | --- | --- | --- | --- | --- |
| Coxarthrosisis | .3937373 | .5233329 | 0.75 | 0.452 | -.6343832 | 1.421.858 |
| Fibromyalgia | 1.423.446 | .4958801 | 2.87 | 0.004 | .4492581 | 2.397.634 |
| Knee arthrosis | .4721019 | .4995804 | 0.94 | 0.345 | -.5093554 | 1.453.559 |
| Lumbar Pain | 1.296.187 | .5009316 | 2.59 | 0.010 | .3120753 | 2.280.299 |
| Shoulder Pain | .8055233 | .5445026 | 1.48 | 0.140 | -.2641864 | 1.875.233 |
| Non cancer chronic pain | .8925018 | .7439322 | 1.20 | 0.231 | -.5689999 | 2.354.004 |
| Arthritis rheumatoid | 9.081.457 | .4758206 | 19.09 | 0.000 | 8.146.678 | 1.001.624 |

Table 4. Average cost by patient drug consumption by diagnostic during the intervention period. (in USD) (1 USD = 952.38 CLP).

| Etiquetas de fila | Rheumatoid Arthritis | Coxarthrosisis | Fibromyalgia | Knee arthrosis | Lumbar Pain | Shoulder Pain | Non cancer chronic pain |
| --- | --- | --- | --- | --- | --- | --- | --- |
| Amitriptilina Clorhidrato 25 Mg Capsules | 93.3 | 360.0 | 2,092.8 | 130.3 | 609.7 | 494.1 | 0.0 |
| Buprenorphine patches 35 mcg/h | 914.9 | 4,494.9 | 9,977.5 | 5,927.3 | 15,271.7 | 11,251.1 | 5,765.3 |
| Celecoxib 200 Mg Capsules | 40.6 | 87.2 | 194.7 | 268.8 | 184.3 | 313.5 | 356.9 |
| Ciclobenzaprina Clorhidrato 10 mg Capsules | 0.0 | 0.0 | 2.6 | 19.0 | 1.6 | 0.0 | 0.0 |
| Diclofenac Sodium 75 Mg/ 3ml Amp | 44.7 | 93.1 | 276.1 | 345.2 | 365.0 | 383.6 | 134.0 |
| Duloxetine 30 mg Capsules | 1,088.9 | 3,096.4 | 23,292.3 | 4224.1 | 12341.5 | 4,828.6 | 1,788.9 |
| Ibuprofen 400 Mg Capsules | 186.1 | 246.2 | 298.8 | 289.3 | 409.0 | 314.0 | 67.0 |
| Metamizol Sodico Injectable Solution1 G/2 Ml | 0.0 | 16.6 | 14.7 | 22.0 | 23.8 | 29.2 | 0.0 |
| Paracetamol 500 Mg Capsules | 216.0 | 47.7 | 265.7 | 287.5 | 129.9 | 221.6 | 0.0 |
| Prednisone 20 Mg Capsules | 1,615.4 | 7,864.7 | 4,704.0 | 8,180.3 | 6,303.8 | 4,853.2 | 2,985.9 |
| Tramadol Clorhidrato 50 Mg Capsules | 93.3 | 6.5 | 17.9 | 62.9 | 0.0 | 2.5 | 0.0 |
| Diclofenac Sodium 50 Mg Capsules | 130.0 | 129.2 | 250.1 | 98.4 | 265.9 | 263.8 | 520.0 |
| Metamizol Sodium 300 Mg Capsules | 7,186.7 | 8,536.0 | 52,759.2 | 11,728.4 | 43,318.2 | 38050.1 | 28,746.7 |
| Prednisone 5 Mg Capsules | 128.1 | 333.4 | 111.6 | 59.6 | 4,291.3 | 187.8 | 0.0 |
| Pregabaline 75 mg Capsules | 21.9 | 399.8 | 401.5 | 245.0 | 1,370.3 | 325.0 | 65.8 |
| Tramadol Clorhidrato Solution 100 Mg/Ml (10 ml) | 0.0 | 2,645.5 | 0.0 | 1,404.8 | 5413.0 | 0.0 | 0.0 |
| **Total consumption** | **11,760.8** | **28,359.1** | **94,662.3** | **33,296.9** | **90,304.1** | **61,524.0** | **40,437.4** |

Figure 1. Distribution of the average cost by patient drug consumption by diagnostic during the intervention period.


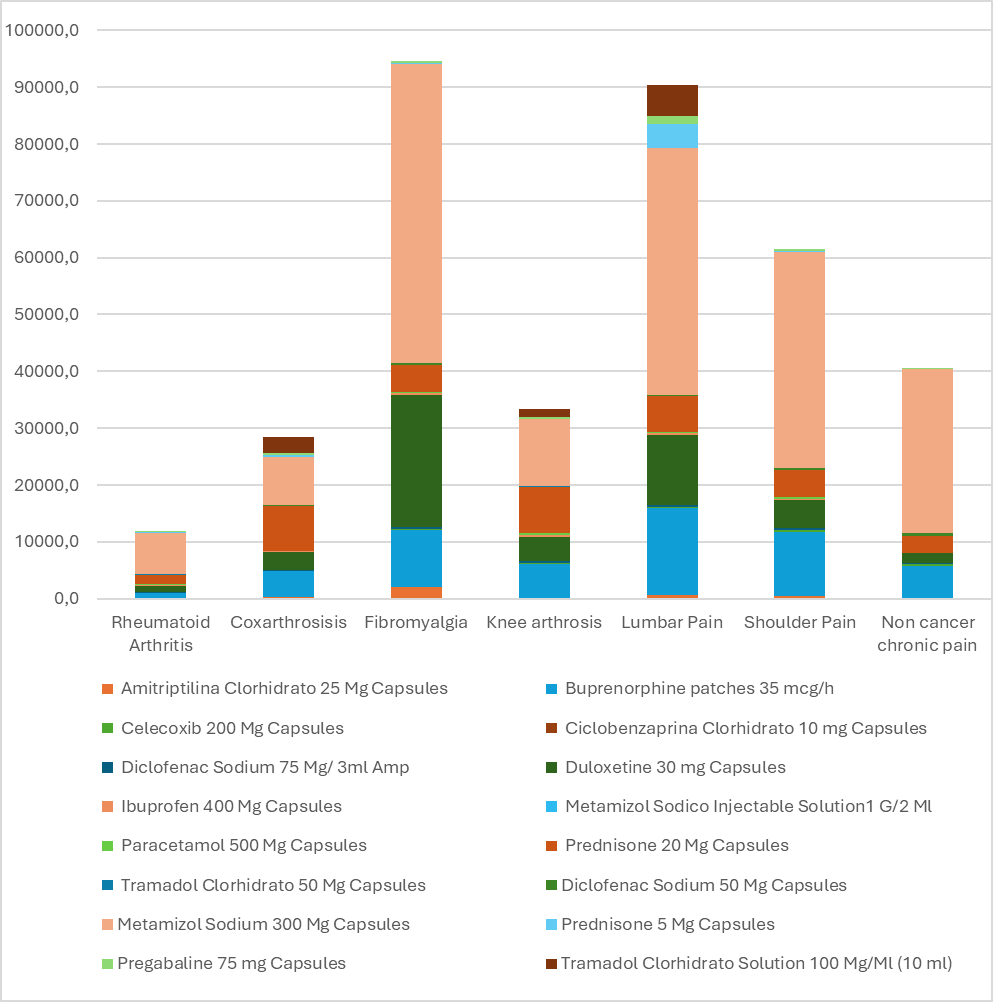

Supplement: Supplementary file 1 — Supplementary Material 1 [file 12913_2025_12560_MOESM1_ESM.doc]
